# *BRAF* and *RAS* mutations in Vietnamese patients with ameloblastoma

**DOI:** 10.1186/s12903-026-08325-3

**Published:** 2026-04-13

**Authors:** Kien Ai Hoang, Chi Thi Kim Nguyen, Chuong Quoc Ho, Tram Bich Duong, Thao Thi Thu Luu, Tam Thi Thanh Bui, Nam Cong Nhat Huynh, Hoang Anh Vu, Tuyen Dac Vo

**Affiliations:** 1 Department of Oral and Maxillofacial Surgery, Odonto-Maxillo-Facial Hospital of Ho Chi Minh City, Ho Chi Minh City, Vietnam; 2 Faculty of Dentistry, University of Medicine and Pharmacy at Ho Chi Minh City, Ho Chi Minh City, Vietnam; 3 Center for Molecular Biomedicine, University of Medicine and Pharmacy at Ho Chi Minh City, Ho Chi Minh City, Vietnam; 4 Department of Histology, Embryology and Pathology, School of Medicine, University of Medicine and Pharmacy at Ho Chi Minh City, Ho Chi Minh City, Vietnam; 5 Department of Oral Pathology, Faculty of Dentistry, University of Medicine and Pharmacy at Ho Chi Minh City, 652 Nguyen Trai Street, Cho Lon Ward, Ho Chi Minh City, 749000 Vietnam

**Keywords:** Ameloblastoma, *BRAF* mutation, *RAS* mutation, *BRAF*^V600E^, MAPK pathway, Vietnam

## Abstract

**Background:**

Ameloblastoma is a benign but locally aggressive odontogenic tumor. Genetic mutations in the mitogen-activated protein kinase (MAPK) signaling pathway, particularly those in *BRAF* and *RAS*, have been increasingly implicated in its pathogenesis, but remain underreported in Vietnamese patients.

**Methods:**

Ninety-six formalin-fixed paraffin-embedded (FFPE) ameloblastoma samples from Vietnamese patients were retrospectively analyzed. Targeted exons of *BRAF* and *RAS* genes were amplified by PCR and sequenced using Sanger sequencing on an ABI 3500 Genetic Analyzer. Mutation frequencies, the prevalence of the *BRAF* p.Val600Glu (*BRAF*^V600E^), and their associations with clinical, radiographic, and histopathological features were evaluated.

**Results:**

MAPK pathway mutations were identified in 63.5% of cases. *BRAF*^V600E^ was the most frequent alteration, detected in 52.1% (50/96) of tumors. *BRAF*^V600E^ mutations were significantly associated with radiographic radiolucent patterns, and were more common in female than in male patients (*p* < 0.05).

In addition, a rare *HRAS* variant, c.22G > A (p.Val8Met) *(HRAS*^V8M^*)*, was identified. To our knowledge, this variant has not been previously reported in ameloblastoma or in public mutation databases and is therefore considered a novel, exploratory finding.

**Conclusions:**

*BRAF* and *RAS* mutations were identified in Vietnamese patients with ameloblastoma, with *BRAF*^V600E^ mutation accounting for more than half of the cases. The significant association of *BRAF* p.Val600Glu with distinct radiographic features and female gender supports its potential value as a biomarker. In addition, the identification of a novel *HRAS* mutation warrants the need for further investigation.

## Background

Ameloblastoma is one of the most common odontogenic tumors; however, its reported prevalence varies widely across studies from different countries, ranging from 25.3% to 75.9% [1–4]. In Vietnam, ameloblastoma accounts for 57.3% of odontogenic tumors [5]. Delayed clinical presentation leads to progressive facial deformity, marked osteolysis with cortical perforation of the mandibular or maxillary periosteum, and subsequent invasion into contiguous anatomical structures. In cases of large tumors, patients may develop marked facial disfigurement, impaired mastication, swallowing, and speech, and in rare instances, ameloblastoma can even be life-threatening [6]. Surgery remains the mainstay of treatment; however, it is often accompanied by significant osseous defects, and in children or elderly patients with comorbidities, surgery may not represent the optimal therapeutic option.

Advancements in sequencing technologies following the Human Genome Project have made genomic analysis more efficient, thereby facilitating investigation of genetic susceptibility and tumor pathogenesis [7]. From 2014 onwards, several molecular investigations in ameloblastoma have been published, offering new insights into its pathogenesis and management. These studies have highlighted cellular signaling pathways involved in tumorigenesis, among which the MAPK (Mitogen-Activated Protein Kinase) pathway plays a crucial role [8, 9]. Mutations in *BRAF* and *RAS*, which activate the MAPK cascade, have been consistently reported. Of particular note, *BRAF* mutations in exon 15 are the most frequent in ameloblastoma, with the substitution of valine by glutamic acid at codon 600 (*BRAF*^V600E^) in the activation loop accounting for approximately 90% of all *BRAF* mutations [10]. Reported frequencies of *BRAF*^V600E^ mutation vary geographically: 43% in the United States [11], 72.5% in Thailand [12], 82% in Brazil [13], and up to 90% in Korea [14]. In contrast, *RAS* mutations in ameloblastoma occur at lower frequencies, with an estimated prevalence of around 20% as reported in the literature [15].

Despite these advances, systematically collected molecular data on ameloblastoma in Vietnamese patients remain limited. Therefore, this study was conducted to address this gap by establishing a foundational molecular dataset describing the frequency and distribution of *BRAF* and *RAS* mutations in a clinically and histopathologically well-defined cohort of Vietnamese patients. In addition to determining mutation frequencies, we examined the associations between mutation status and clinical, histopathological, and radiological features in order to provide a comprehensive descriptive framework for the interpretation of molecular findings.

## Methods

### Study design

This descriptive cross-sectional study investigated *BRAF* and *RAS* mutations in ameloblastomas, and assessed their associations with clinical, radiographic and histopathological features.

The study including patients diagnosed with ameloblastoma who were examined and treated at the Department of Oral and Maxillofacial Surgery, Ho Chi Minh City Hospital of Odonto-Stomatology, from January 2021 to January 2025.

#### Inclusion criteria 

Histopathologically confirmed diagnosis of ameloblastoma; availability of complete medical records; availability of FFPE tissue blocks, and informed consent to participate in the study.

#### Exclusion criteria 

No additional exclusion criteria were applied.

The minimum sample size was calculated using the formula for estimating a proportion:

${\text n}\geq\left(\mathrm{Z}\left(1-\alpha /2\right)/{\text d}\right)^2\;.\mathrm{p}\;.\left(1-{\text p}\right)$. With α = 0.05 (95% confidence level), $\mathrm{Z}\left(1-\alpha/2\right)=1.96$, p was estimated from previous studies, and d = 0.1 represents the margin of error (absolute precision).

Based on published data, the mutation frequency of *BRAF* in ameloblastoma was 46% [11] and that of *RAS* was 20% [15]. The corresponding minimum sample size required was 96 cases for *BRAF* and 62 cases for *RAS*. Therefore, the larger value (96) was adopted as the target sample size for this study.

### Data collection

Clinical variables (age, sex, and tumor status: primary or recurrent) were retrieved from medical records. Primary status was defined as newly diagnosed ameloblastoma at first presentation, whereas recurrence status referred to patients presenting with a recurrent ameloblastoma after previous surgery, not recurrence observed during prospective follow-up. Radiographic characteristics were evaluated on panoramic radiographs obtained with a Planmeca ProMax 3D unit (Planmeca Oy, Helsinki, Finland) and on computed tomography (CT) scans acquired using a Toshiba Aquilion 640 or Canon Aquilion ONE PRISM 640 system (Canon Medical Systems, Japan), with images reviewed on the integrated Vitrea portable viewer. Radiographic features were assessed using predefined and standardized criteria. Tumor location was recorded according to anatomical subsite, including the maxilla (three classifications: anterior maxilla from cuspid to cuspid, posterior maxilla from the premolar region to the pterygoid plates, and lesions involving both anterior and posterior maxillary regions) and the mandible (three classifications: symphysis–body, angle–ramus, and lesions extending from the symphysis through the angle to the ramus) [16]; lesion margin (well-defined with sclerosis, well-defined without sclerosis, or ill-defined) [17]; radiolucent pattern (unilocular or multilocular, with multilocular lesions exhibiting soap-bubble, honeycomb, or spider-web appearance) [18], cortical bone status (expansion, perforation) [18], type of root resorption (knife-edge, spike, or multiplanar type) [18], and association with impacted teeth [18]. Histopathological data were reviewed on hematoxylin and eosin (H&E) stained-slides, and ameloblastomas were classified according to the 2017 WHO Classification of Head and Neck Tumours. Ameloblastomas were classified into two gross types: unicystic and conventional. Unicystic ameloblastoma included the luminal, intraluminal, and mural subtypes, whereas conventional ameloblastoma comprised the follicular, plexiform, acanthomatous, granular cell, basal cell, desmoplastic, and mixed variants.

All radiographs and histopathological slides were independently evaluated by two investigators. Inter-observer agreement was assessed using Cohen’s κ statistic, and discrepancies were resolved by consensus review.

Mutation data were proceeded in the following steps:

#### Primer design for PCR and sequencing

Reference sequences of *BRAF* (NG_007873, NM_004333), *KRAS* (NG_007524, NM_004985), *NRAS* (NG_007572, NM_002524), and *HRAS* (NG_007666, NM_005343) were retrieved from the GenBank database (NCBI). Primer pairs targeting frequently mutated regions were designed using CLC Main Workbench v5.5. The primer physical properties were evaluated with Oligo Analyzer 3.1 (Integrated DNA Technologies). Synthesized primers were purchased from Integrated DNA Technologies (IDT, USA). The primer sequences are listed in Table 1. After primer design, annealing temperatures between 54 °C and 62 °C were evaluated. PCR reactions were performed for each primer pair to identify the optimal annealing temperature that produced specific and efficient amplification.

**Table 1:** Primer sequences used for amplification of target gene regions

Gene	Exon	Primer name	Primer sequence (5’ – 3’)	Annealing temperature (^0^C)	Product size (bp)
*BRAF*	11	BRAF-g11F2	TTTCTGTTTGGCTTGACTTG	56	273
		BRAF-g11R	GTTTATTGATGCGAACAGTG		
	15	BRAF-600 F	ACTCTTCATAATGCTTGCTC	55	184
		BRAF-600R	CCACAAAATGGATCCAGACA		
*KRAS*	2	KRAS-E2F	AGGCCTGCTGAAAATGACTGAATA	55	178
		KRAS-E2R	CTGTATCAAAGAATGGTCCTGCAC		
	3	KRAS-61 F	TTCTCAGGATTCCTACAGGA	56	194
		KRAS-61R	AAACCCACCTATAATGGTGA		
*NRAS*	2	NRAS-1 F	GATGTGGCTCGCCAATTAAC	55	232
		NRAS-1R	GGTAAAGATGATCCGACAAGTG		
	3	NRAS-61 F	ACCCCCAGGATTCTTACAGA		197
		NRAS-61R	CTCCTAGTACCTGTAGAGGT		
*HRAS*	2	HRAS-2 F	CAGGAGACCCTGTAGGAGGA	58	139
		HRAS-2R	TCGTCCACAAAATGGTTCTG		
	3	HRAS-3 F	ATTCCTACCGGAAGCAGGTG		156
		HRAS-3R	AAGACTTGGTGTTGTTGATG		

5’ – 3’: Primer sequences are presented in the 5′–3′ direction

*bp* base pairs

#### DNA extraction from FFPE tissue

Genomic DNA was extracted from FFPE tissue blocks using the ReliaPrep™ FFPE gDNA Miniprep System (Promega, USA) according to the manufacturer’s instructions, yielding 50 µL of gDNA solution. DNA purity was measured with a NanoDrop 2000 spectrophotometer (Thermo Fisher Scientific, USA). Extracted DNA was stored at − 20 °C until further use.

Given the known fragmentation and chemical modification of DNA derived from FFPE tissues, downstream analyses were restricted to short amplicons targeting well-established hotspot regions. DNA quality was initially assessed by spectrophotometric measurement; however, we acknowledge that NanoDrop-based purity assessment does not fully reflect DNA integrity. Samples that failed to yield a clear, single PCR product of the expected size were excluded from further analysis. To minimize the risk of FFPE-related artifacts, all variants were confirmed by bidirectional Sanger sequencing and reproducibility was verified using independent PCR amplifications. In addition, histopathological review was performed prior to DNA extraction to exclude samples with insufficient tumor cellularity.

#### PCR amplification

Target regions of the selected genes were amplified using specifically designed primer pairs. The reaction mixture (15 µL total volume) consisted of 1.5 µL 10× PCR buffer, 1.5 µL dNTPs (2.5 mM), 0.75 µL forward primer (10 µM), 0.75 µL reverse primer (10 µM), 0.1 µL TaKaRa Taq HS polymerase (Takara Bio, Japan), 9.4 µL nuclease-free water, and 1 µL gDNA template. Thermal cycling conditions were optimized for each primer set. The hot-start property of Taq polymerase, achieved by binding a monoclonal antibody, minimized nonspecific priming and reduced primer–dimer formation. Amplification success was confirmed when a clear single band of the expected size was obtained without nonspecific amplification.

#### Gel electrophoresis

PCR products were separated on 1% agarose gels prepared in 0.5× TBE buffer, alongside a 1 kb Plus DNA ladder (Thermo Fisher Scientific, USA) as a size reference. Electrophoresis was performed at 100 V for 30 min.

#### PCR product purification

Amplified products were purified using the ExoSAP-IT^®^ PCR Product Cleanup Reagent (Applied Biosystems, USA). Each reaction contained 5 µL PCR product, 1 µL ExoSAP-IT, and 1 µL nuclease-free water. Samples were incubated at 37 °C for 15 min, followed by enzyme inactivation at 80 °C for 15 min.

#### Sequencing reactions

Purified PCR products were sequenced using the BigDye^®^ Terminator v3.1 Cycle Sequencing Kit (Applied Biosystems, USA) with both forward and reverse primers (bidirectional Sanger sequencing). Each 9.5 µL reaction contained 0.5 µL BigDye Terminator v3.1 (2.5×), 1.5 µL sequencing buffer (5×), 2 µL primer (1.6 µM), 1 µL purified PCR product, and 4.5 µL nuclease-free water. Cycling conditions consisted of initial denaturation at 96 °C for 1 min; 30 cycles of 96 °C for 10 s, 50 °C for 5 s, and 60 °C for 4 min; and a final hold at 4 °C. Sequencing primers were manually designed in CLC Main Workbench v5.5 (CLC bio, Denmark) based on validated PCR products.

#### Sequencing product precipitation

Sequencing products were precipitated with ammonium acetate and ethanol prior to electrophoresis. Briefly, 12 µL of 5 M NH_4_OAc and 60 µL of 100% ethanol were added to each sample, mixed thoroughly, and transferred to 1.5 mL microcentrifuge tubes. Samples were centrifuged at 14,600 rpm for 20 min at 4 °C, and the supernatant was discarded. The pellet was washed with 400 µL of 70% ethanol and centrifuged again at 14,600 rpm for 25 min at 4 °C. After vacuum drying at 48 °C, the pellet was resuspended in 17 µL Hi-Di formamide (Applied Biosystems, USA), vortexed, and briefly centrifuged. Samples were then denatured at 96 °C for 2 min, cooled on ice, and stored at − 30 °C for 5 min before loading into a 96 well sequencing plate.

#### Sequencing and data analysis

Capillary electrophoresis was performed on an ABI 3500 Genetic Analyzer (Applied Biosystems, USA). Raw sequence data were analyzed with CLC Main Workbench v5.5. Consensus sequences were aligned with reference sequences of *BRAF*,* KRAS*,* NRAS*,* and HRAS* obtained from GenBank (NCBI) to identify mutations.

### Statistical analysis

Data were entered and analyzed using SPSS version 22.0, and ODAS software. Categorical variables (sex, age groups, radiographic features, histological types) were summarized as frequencies and percentages. Continuous variables were expressed as mean ± standard deviation (SD) for normally distributed data or as median and interquartile range (IQR) for non-normally distributed data.

Associations between gene mutations and clinicopathological parameters were assessed using the Chi-square test. Fisher’s exact test was applied when more than 20% of expected cell counts were < 5 or when any expected count was < 1. A p-value < 0.05 was considered statistically significant.

## Results

### Clinicopathological characteristics of the study cohort

A total of 96 patients with ameloblastoma were included, comprising 42 males and 54 females (male-to-female ratio = 0.78:1). The mean age at diagnosis was 32.2 years (SD 16.3; range 8–69 years) with the most common age group being 20–39 years (44.8%). The mandible was the most frequently affected site (89.6%). Most tumors were primary lesions, whereas only five cases (5.2%) represented recurrences. Conventional ameloblastomas accounted for a greater proportion than the unicystic variant (79.2% vs. 20.8%). The distribution of clinical and gross features according to histological type is presented in Table 2, with no statistically significant differences were observed.

**Table 2:** Distribution of ameloblastoma cases by clinicopathological features

Characteristic	Total	Unicystic type	Conventional type	*p*- value
	*N* = 96	*N* = 20	*N* = 76	
Age group				
0–19 years	26 (27.1)	7 (26.9)	19 (73.1)	0.399^a^
20–39 years	43 (44.8)	8 (18.6)	35 (81.4)	
40–59 years	19 (19.8)	5 (26.3)	14 (73.7)	
≥ 60 years	8 (8.3)	0 (0.0)	8 (100.0)	
Sex				
Female	54 (56.3)	14 (25.9)	40 (74.1)	0.164^b^
Male	42 (43.8)	6 (14.3)	36 (85.7)	
Tumor status				
Primary	91 (94.8)	18 (19.8)	73 (80.2)	0.278^a^
Recurrent	5 (5.2)	2 (40.0)	3 (60.0)	
Tumor location				
Maxilla	10 (10.4)	3 (30.0)	7 (70.0)	0.430^a^
Mandible	86 (89.6)	17 (19.8)	69 (80.2)	

^a^ Fisher’s exact test

^b^ Chi-square test

### Frequency and mutation patterns of BRAF and RAS genes in the MAPK pathway

**Fig. 1:**
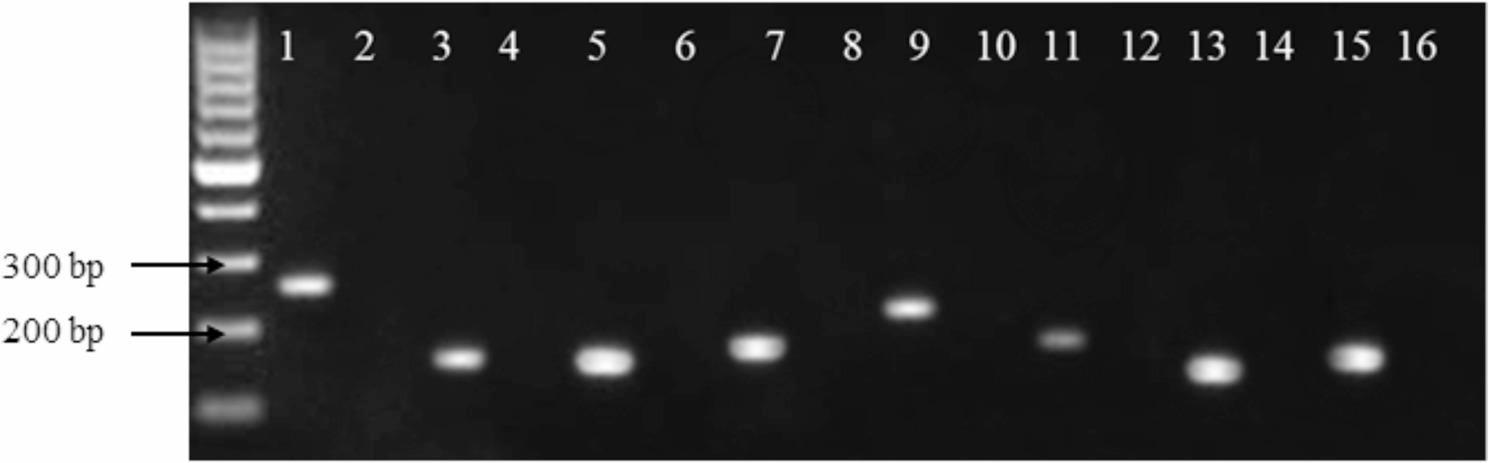
Gel electrophoresis of PCR products from *BRAF* and *RAS* genes. Wells 1: *BRAF* exon 11; Wells 3: *BRAF* exon 15; Wells 5: *KRAS* exon 2; Wells 7: *KRAS* exon 3; Wells 9: *NRAS* exon 2; Wells 11: *NRAS* exon 3; Wells 13: *HRAS* exon 2; Wells 15: *HRAS* exon 3. H₂O (negative control): wells 2, 4, 6, 8, 10, 12, 14, and 16

Amplification of the target regions of *BRAF* and *RAS* genes was performed using specifically designed primer pairs, and the resulting PCR products were subsequently resolved by electrophoresis on 1% agarose gels (Fig. 1).

Mutations in the MAPK pathway were identified in 61 of 96 cases (63.5%). Alterations in the *BRAF* gene were found in 55 cases (57.3%), whereas *RAS* mutations were detected in 6 cases (6.3%). A total of nine distinct mutation types were identified. Most alterations occurred as single mutations, with the *BRAF*^V600E^ substitution being predominant, detected in 50 cases (52.1%) and representing 90.9% of all *BRAF* mutations (Table 3).

**Table 3:** Frequency and mutation patterns of *BRAF* and *RAS* genes in the MAPK pathway

Mutation type	Frequency *N* (%)
MAPK pathway mutations	61 (63.5)
*BRAF* mutations	55 (57.3)
Exon 11: c.1333G > A (D445N), c.1375G > A (K459M), c.1397 G > A (G466E)	3 (3.1)
Exon 15: c.1798G > A (V600M), c.1828T > C (F610L)	2 (2.1)
Exon 15: c.1799T > A (V600E)	50 (52.1)
*RAS* mutations	6 (6.3)
*KRAS* (exon 2): c.35G > T (G12V), c.50G > A (S17N)	2 (2.1)
*NRAS* (exon 2): c.34G > A (G12S)	1 (1.0)
*HRAS* (exon 2): c.31G > A (A11T), c.22G > A (V8M)	2 (2.1)
*HRAS* (exon 3): c.205G > A (D69N)	1 (1.0)

**Fig. 2:**
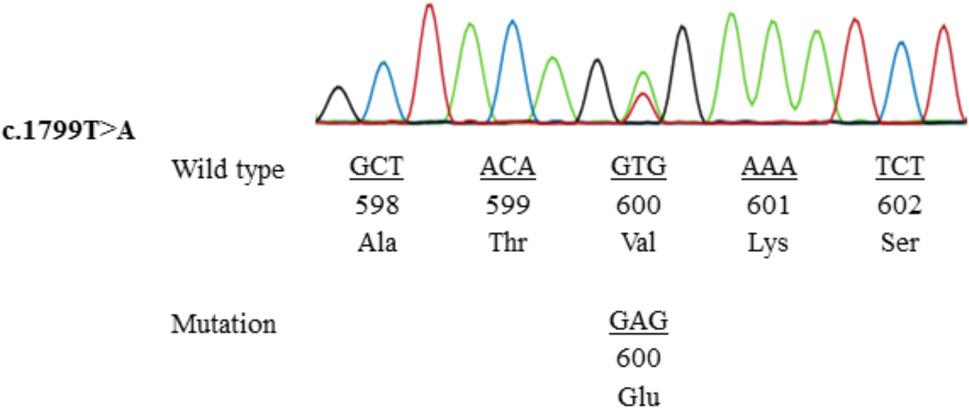
Mutation c.1799T > A (p.Val600Glu) in exon 15 codon 600 of *BRAF* gene (*BRAF*^V600E^) (50/96 cases)

The *BRAF*^V600E^ mutation identified in the study is a point mutation in which thymine (T) at nucleotide position 1799 is replaced by adenine (A) (c.1799T > A) (Fig. 2).

**Fig. 3:**
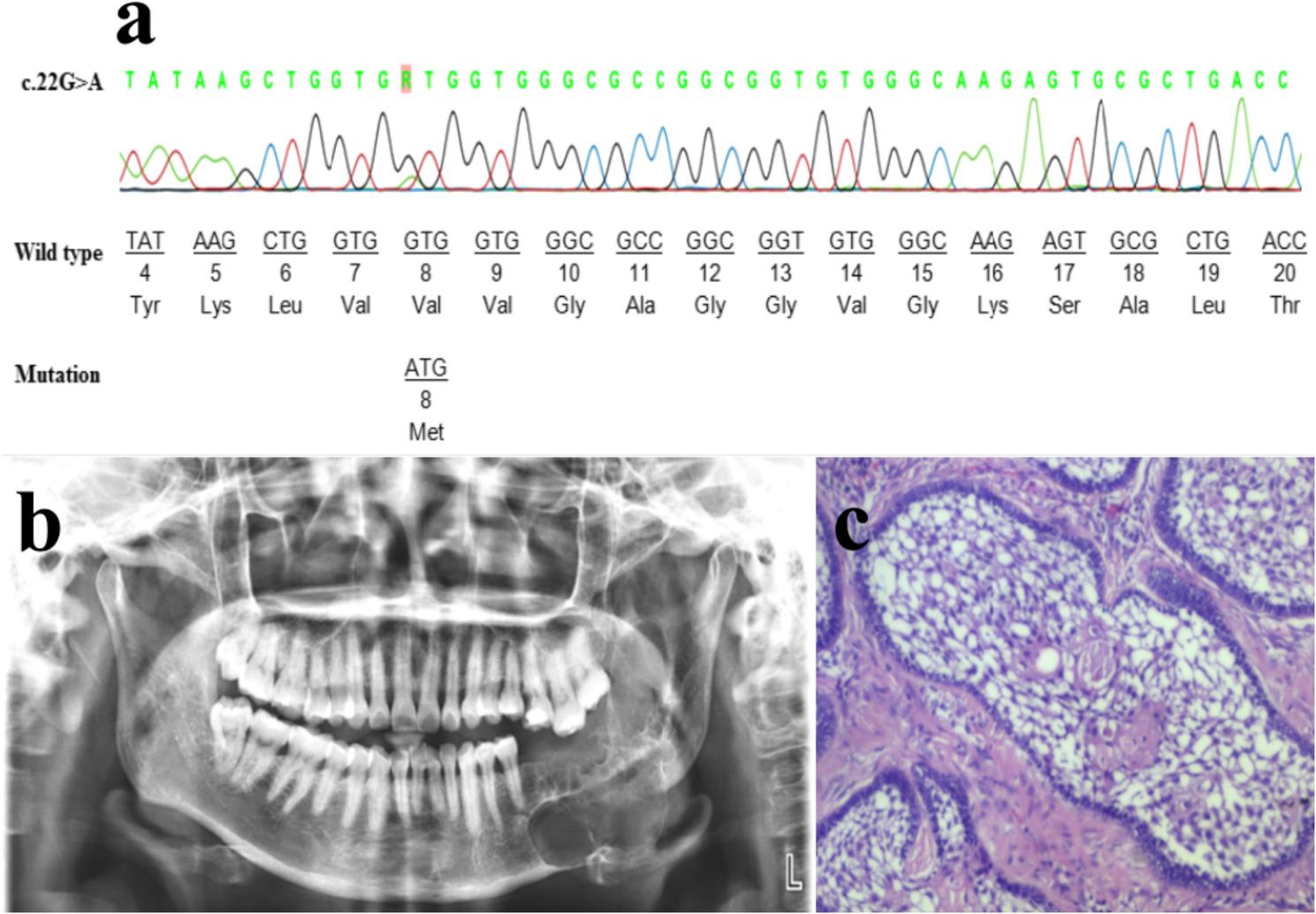
Mandibular ameloblastoma *with HRAS*^V8M^ mutation (case 3): (**a**) Mutation c.22G > A (p.Val8Met) in exon 2 codon 8 of *HRAS*, (**b**) Radiograph showing a soap-bubble radiolucent lesion with well-defined, non-sclerotic margins, and (**c**) Acanthomatous histological pattern (×40)

In addition, an *HRAS* variant, c.22G > A (p.V8M), was detected in case 3, characterized by a soap-bubble radiolucent lesion with well-defined, non-sclerotic margins and an acanthomatous histological pattern (Fig. 3).

### Association between BRAF and RAS mutations in the MAPK pathway and clinicopathological and radiographic features

The mean age in mutation-positive and mutation-negative groups ranged from 31 to 34 years, with no statistically significant difference between them (Table 4).

**Table 4:** Mean age of patients according to mutation status

	Mean age (years)	Standard deviation (SD)	*p*-value
MAPK pathway mutations			
Absent	33.2	18	0.635
Present	31.6	15.4	
*BRAF*^V600E^ mutation			
Absent	31.6	17.3	0.745
Present	32.7	15.5	

Independent samples t-test assuming equal variances

MAPK pathway mutation was defined as at least one mutation in *BRAF* or *RAS*

The highest frequency of MAPK pathway mutations was observed in the 40–59 year age group (68.4%). Mutation rates were higher in recurrent tumors compared with primary tumors for both overall MAPK pathway mutations and specifically for *BRAF*^V600E^ (80% vs. 60%, respectively), although these differences were not statistically significant. Female patients exhibited a significant higher prevalence of *BRAF*^V600E^ mutations compared with males (61.1% vs. 40.5%) (Fisher’s exact test, *p* = 0.045) (Table 5).

**Table 5:** Association between gene mutations and clinicopathological features

Characteristic	Total *N* (%)	MAPK pathway mutations		BRAF^V600E^ mutation	
		Present *N* (%)	*p*-value	Present *N* (%)	*p*-value
Age group					
0–19 years	26 (27.1)	15 (57.7)	0.681^a^	11 (42.3)	0.291^a^
20–39 years	43 (44.8)	29 (67.4)		23 (53.5)	
40–59 years	19 (19.8)	13 (68.4)		13 (68.4)	
≥ 60 years	8 (8.3)	4 (50.0)		3 (37.5)	
Sex					
Female	54 (56.3)	37 (68.5)	0.251^b^	33 (61.1)	0.045^b^
Male	42 (43.8)	24 (57.1)		17 (40.5)	
Tumor status					
Primary	91 (94.8)	57 (62.6)	0.650^a^	47 (51.6)	0.999^a^
Recurrent	5 (5.2)	4 (80.0)		3 (60.0)	

MAPK pathway mutation was defined as at least one mutation in *BRAF* or *RAS*

^a^ Fisher’s exact test

^b^ Chi-square test

In the MAPK pathway, mutations were more frequent in mandibular than in maxillary tumors (65.1% vs. 50.0%). Within the mandible, the highest frequency occurred in the symphysis–body region (66.7%). Mutations tended to be more common in ameloblastomas exhibiting a soap-bubble pattern, well-defined without sclerotic margins, absence of impacted teeth, spike type root resorption, and cortical expansion; however, these associations did not reach statistical significance.

**Fig. 4:**
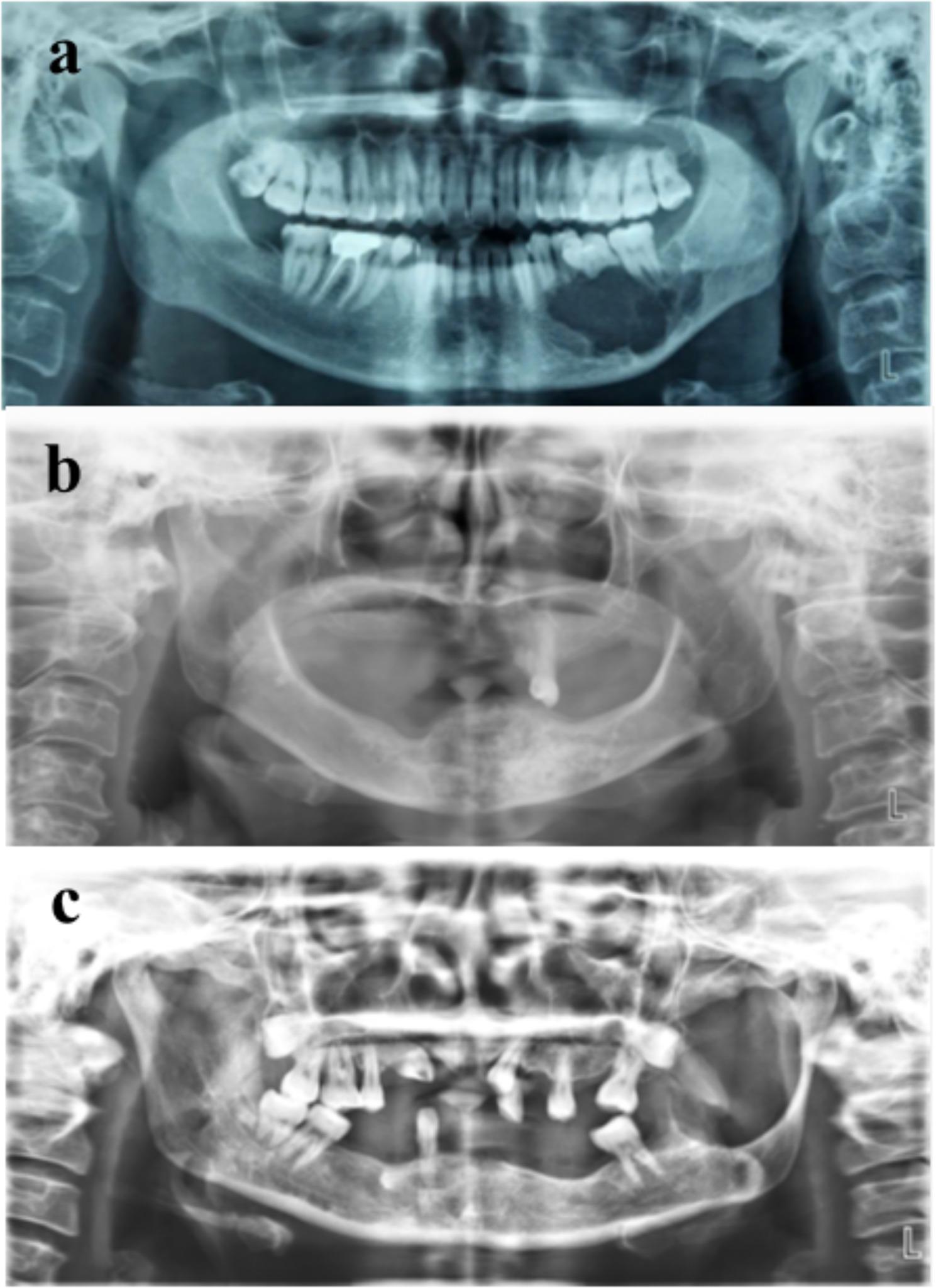
Radiographic features of mandibular ameloblastomas with different *BRAF* mutations. **a** Case 83: *BRAF*^V600E^ mutation, soap-bubble radiolucency with well-defined, without sclerotic margins in the left mandibular body. **b** Case 21: *BRAF*^D445N^ mutation, honeycomb radiolucency with ill-defined margins in the symphysis region. **c** Case 58: *BRAF*^V600E^ mutation, unilocular radiolucency with well-defined and sclerotic margins in the left mandibular ramus

*BRAF*^V600E^ mutations were also more prevalent in mandibular than in maxillary tumors (54.7% vs. 30.0%), with the highest prevalence in the angle–ramus region (59.3%). Notably, *BRAF*^V600E^ mutations were significantly associated with radiolucent patterns (soap-bubble and unilocular) (*p* = 0.040). (Table 6; Fig. 4).

**Table 6:** Association between gene mutations and radiographic features

Feature	Total *N* (%)	MAPK pathway mutations		BRAF^V600E^ mutation	
		Present *N* (%)	*p*-value	Present *N* (%)	*p*-value
Tumor location					
Maxilla	10 (10.4)	5 (50.0)	0.489^a^	3 (30.0)	0.187^a^
Mandible	86 (89.6)	56 (65.1)		47(54.7)	
Tumor region					
Anterior maxilla	1 (1.0)	1 (100)	0.843^a^	0 (0)	0.731^a^
Anterior–posterior maxilla	7 (7.3)	3 (42.9)		2 (28.6)	
Posterior maxilla	2 (2.1)	1(50.0)		1 (50.0)	
Symphysis–body	48 (50.0)	32 (66.7)		25 (52.1)	
Angle- ramus	27 (28.1)	17 (63.0)		16 (59.3)	
Symphysis–angle–ramus	11 (11.5)	7 (63.6)		6 (54.5)	
Radiolucent pattern					
Unilocular	47 (49.0)	29 (61.7)	0.439^a^	24 (51.1)	0.040^a^
Soap-bubble	44 (45.8)	30 (68.2)		26 (59.1)	
Honeycomb	5 (5.2)	2 (40.0)		0 (0)	
Margin					
Ill-defined	23 (24.0)	13 (56.5)	0.575^b^	10 (43.5)	0.234^b^
Well-defined, sclerotic	31 (32.3)	19 (61.3)		14(45.2)	
Well-defined, Without sclerotic	42 (43.8)	29 (69.0)		26 (61.9)	
Impacted tooth					
Absent	65 (70.8)	43 (66.2)	0.441^b^	35 (53.8)	0.617^b^
Present	31 (32.3)	18 (58.1)		15 (48.4)	
Root resorption					
Absent	51 (53.1)	33 (64.7)	0.640^a^	28 (54.9)	0.520^a^
Knife-edge	33 (34.4)	19 (57.6)		14 (42.4)	
Multi-planar	9 (9.4)	6 (66.7)		6 (66.7)	
Spike	3 (3.1)	3 (100)		2 (66.7)	
Cortical bone alterations					
None	3 (3.1)	1 (33.3)	0.206^a^	1 (33.3)	0.501^a^
Expansion	45 (46.9)	32 (71.1)		26 (57.8)	
Perforation	48 (50.0)	28 (58.3)		23 (47.9)	

^a^ Fisher’s exact test

^b^ Chi-square test

MAPK pathway mutation was defined as at least one mutation in *BRAF* or *RAS*

MAPK pathway mutations, including *BRAF*^V600E^, were more frequent in unicystic than conventional ameloblastomas (75% vs. 60.5% and 70% vs. 47.4%, respectively). By histological subtype, MAPK mutations were highest in the luminal subtype (80%), while *BRAF*^V600E^ occurred most often in mural and luminal subtypes (70%). In conventional tumors, the acanthomatous subtype showed the highest MAPK mutation rate (66.7%), and the plexiform subtype exhibited the highest *BRAF*^V600E^ frequency (58.8%). However, none of these correlation were statistically significant (Table 7).

**Table 7:** Association between gene mutations and histopathological features

Feature	Total *N* (%)	MAPK pathway mutations		BRAF^V600E^ mutation	
		Present *N* (%)	*p*-value	Present *N* (%)	*p*-value
Gross type					
Unicystic	20 (20.8)	15 (75.0)	0.231^a^	14 (70.0)	0.071^a^
Conventional	76 (79.2)	46 (60.5)		36 (47.4)	
Histological subtype					
Luminal	10 (10.4)	8 (80.0)	0.847^b^	7 (70.0)	0.364^b^
Mural	10 (10.4)	7 (70.0)		7 (70.0)	
Mixed	42 (43.8)	25 (59.5)		20 (47.6)	
Plexiform	17 (17.7)	11 (64.7)		10 (58.8)	
Follicular	5 (5.2)	3 (60.0)		2 (40.0)	
Acanthomatous	9 (9.4)	6 (66.7)		4 (44.4)	
Desmoplastic	3 (3.1)	1 (33.3)		0 (0)	

MAPK pathway mutation was defined as at least one mutation in *BRAF* or *RAS*

^a^ Chi-square test

^b^ Fisher’s exact test

## Discussion

Since 2014, several studies have established the MAPK signaling pathway as the main pathogenic mechanism in ameloblastoma [10, 15, 19, 20]. The *RAS* family (*KRAS*, *NRAS*, *HRAS*) and *BRAF* genes are positioned upstream in the cascade and initiate the entire RAS–BRAF–MEK–ERK signaling pathway. Mutations in these genes are sufficient to constitutively activate MAPK signaling, leading to abnormal proliferation and tumor formation [9, 21]. Other genes in the MAPK pathway, such as MEK and ERK, have not yet been clinically validated. Therefore, in the present study, we focused on *BRAF* and *RAS* mutations, which are the most biologically and clinically relevant and ensure feasibility.

In our study, the mean patient age was 32.2 years (± 16.3), ranging from 8 to 69 years, with the most common age group being 20–39 years (44.8%). These demographic findings are consistent with previous reports [22–25]. Ameloblastoma showed no sex predilection in earlier studies [23–25], whereas in our series female patients were slightly more common (male-to-female ratio 0.78:1). Mandibular lesions predominated (89.6%), and the conventional type was more frequent than the unicystic type (79.2% vs. 20.8%), consistent with the literature [24, 26, 27]. These findings support the representativeness of our cohort. In this retrospective study, unicystic ameloblastoma was diagnosed based on total enucleation specimens rather than excisional biopsies. Systematic serial sectioning of the entire specimen was not performed within the scope of the present study. Nevertheless, archived paraffin blocks were obtained from the pathology laboratory, and new H&E-stained sections were recut and independently reviewed for histopathological confirmation. This diagnostic approach was considered appropriate for the predefined objectives of the present analysis.

*RAS* mutations in ameloblastoma typically occur in exons 2 and 3 [15, 28], while *BRAF* mutations are most frequently identified in exons 11 and 15 [29]. Interestingly, mutations in these two genes appear to be mutually exclusive, rarely occurring together [11, 15, 20]. Activating mutations affecting genes within the same signaling pathway are often mutually exclusive. In the MAPK/ERK pathway, oncogenic alterations in *RAS* and *BRAF* lead to activation of the same downstream signaling cascade. Concurrent mutations may therefore provide little additional selective advantage because they generate redundant oncogenic signaling. Moreover, excessive MAPK pathway activation resulting from simultaneous mutations can trigger oncogene-induced senescence, limiting the expansion of double-mutant clones. These mechanisms likely explain why *BRAF* and *RAS* mutations are typically observed as alternative rather than co-occurring events in odontogenic tumors, including ameloblastoma [30, 31]. Therefore, in this study, we sequenced *BRAF* exons 11 and 15 first, and in negative cases we proceeded to *RAS* sequencing (*NRAS*, *HRAS*, *KRAS* in exons 2 and 3). Among 96 paraffin-embedded samples, MAPK pathway mutations were identified in 61 cases (63.5%), including 55 *BRAF* mutations (57.3%) and six *RAS* mutations (6.3%). The *RAS* mutation rate was lower than that reported by Brown and Sweeney, who found a prevalence of approximately 20% [11, 15]. A systematic review by Guimarães also confirmed that *RAS* mutations in odontogenic tumors, including ameloblastomas, are uncommon [9]. These findings suggest that *BRAF* mutations represent the predominant driver of the MAPK pathway in ameloblastoma, whereas *RAS* mutations appear less frequent in this tumor type. However, *RAS* mutations remain biologically relevant in odontogenic tumors and have been reported as key molecular events in other entities, particularly adenomatoid odontogenic tumors, where *KRAS* p.Gly12 mutations are highly prevalent [32, 33].

We detected *BRAF*^V600E^ mutations in 50 of 96 cases (52.1%). This prevalence is lower than reported in Asian countries such as Korea (90%) [14], Thailand (72.5%) [12], Japan, and China (> 70%), but comparable to reports in Europe and the United States (40–65%) [34]. Ethnic and population differences may partly explain this variability. Moreover, differences in methodology, such as the use of immunohistochemistry (which may yield false positives) versus PCR-based sequencing (which may miss novel variants), may also influence reported frequencies. Nevertheless, most studies consistently report *BRAF*^V600E^ mutations in more than 50% of cases. In 2020, González et al. reviewed 19 studies involving 611 ameloblastomas and reported a pooled *BRAF*^V600E^ prevalence of 60% (366/611). In that analysis, single *BRAF*^V600E^ mutations without co-occurring variants accounted for 93.7% of positive cases, while multiple mutations were present in only 6.3%. This finding explains why targeted therapies directed against *BRAF*^V600E^, such as vemurafenib or dabrafenib, may be effective [35]. In our cohort, 12 MAPK pathway mutations were identified, predominantly as isolated events. All six *BRAF* variants detected were RAS-independent [36], with *BRAF*^V600E^ accounting for 90.9% of all *BRAF*-mutant cases, consistent with previous reports [29]. Six *RAS* variants were identified across *NRAS*, *HRAS*, and *KRAS* in this cohort. Among them, a novel *HRAS* mutation, c.22G > A (p.Val8Met) *(HRAS* ^V8M^*)*, was detected. The novel mutation occurred in case 3, a 36-year-old female patient with ameloblastoma of the mandibular ramus. The tumor presented as a conventional type with acanthomatous histological pattern, showing a soap-bubble radiolucency, well-defined, non-sclerotic margins, and cortical perforation (Fig. 3). To the best of our knowledge, this variant has not been previously reported in mutation databases. *HRAS* alterations are generally rare in odontogenic tumors, with most molecular studies documenting only sporadic cases at codons 12, 13, or 61 [37]. The identification of *HRAS*^V8M^ therefore expands the mutational spectrum of *HRAS* and provides further evidence of the genetic heterogeneity underlying this neoplasm. Although the biological significance of this substitution remains to be determined, its location within the N-terminal region of *HRAS* suggests a potential role in altering protein conformation and driving aberrant activation of the MAPK/ERK signaling cascade. Similar early-codon *RAS* variants have been implicated in oncogenesis in other tumor types [38], supporting the hypothesis that this mutation may also act as an activating event. In the present study, sequencing of matched non-tumoral tissue was not performed; therefore, the somatic or germline origin of this variant could not be directly established. Nevertheless, within the histopathological and clinical context of the reported case - including the detection of the variant exclusively in tumor tissue, concordant histomorphological features, and the absence of clinical manifestations suggestive of a hereditary syndrome - the *HRAS*^V8M^variant is interpreted as being most consistent with a somatic alteration. Further functional studies are warranted to elucidate the oncogenic potential of this variant and to determine whether it modulates sensitivity or resistance to targeted therapies currently under investigation for tumors driven by aberrant RAS/MAPK signaling. In addition, because molecular analyses were conducted on FFPE-derived DNA using a hotspot-targeted PCR–Sanger sequencing approach, the potential influence of fixation-related base substitution artifacts cannot be entirely excluded. Accordingly, the HRAS^*V8M*^ variant should be interpreted within these methodological constraints and regarded as hypothesis-generating, pending confirmation using orthogonal sequencing approaches and functional validation. Apart from the case harboring a rare *HRAS* mutation and the recurrent cases described above, the remaining cases with *RAS* family mutations in this cohort demonstrated considerable heterogeneity with respect to both anatomical location and histopathological subtype. Specifically, two maxillary ameloblastomas, corresponding to the luminal and follicular subtypes, were found to harbor *KRAS* mutations. The other two cases occurred in the mandible, including one desmoplastic ameloblastoma with an *NRAS* mutation and one plexiform ameloblastoma with an *HRAS* mutation. These findings indicate that *RAS* mutations in ameloblastoma are not restricted to a single histopathological subtype or anatomical site, but may be encountered across a spectrum of morphological presentations. Notably, the identification of *KRAS* mutations in maxillary ameloblastomas aligns with previously reported observations [20], whereas the *NRAS*- and *HRAS*-mutant tumors in the present cohort were confined to the mandible but exhibited distinct histopathological patterns. Collectively, these observations support the notion that ameloblastoma represents a molecularly heterogeneous neoplasm, in which alterations in RAS pathway genes may contribute to tumorigenesis through diverse biological mechanisms. However, given the limited number of *RAS*-mutant cases in this study, these findings should be interpreted with caution and require confirmation in larger cohorts using more comprehensive genomic profiling strategies.

The mean age of patients with and without MAPK mutations was similar (31–34 years). The 40–59 year group had the highest frequency of mutations (68.4%), but this difference was not statistically significant. This finding differs from Bonacina and Kelppe, who reported lower mean ages in *BRAF*^V600E^-positive cases [39, 40].

Most previous studies found no sex-related difference in *BRAF*^V600E^ prevalence [34, 40, 41]. However, in our series, females had a significantly higher frequency of *BRAF*^V600E^ mutations compared with males (61.1% vs. 40.5%, Fisher’s exact test, *p* = 0.045). This unexpected finding contradicts most of the published literature and raises questions about potential biological or population-specific factors underlying the observed disparity. One plausible hypothesis is the influence of sex hormones on MAPK pathway activity. Experimental studies in breast and thyroid cancer cells have demonstrated that estrogen can activate RAF–MEK–ERK signaling [42, 43]. In addition, investigations in thyroid cancer [44] and cancer in general [45] have also reported a link between estrogen and MAPK pathway activation. These findings suggest that hormonal influences may partly explain the higher frequency of *BRAF*^V600E^ mutations observed in females in our cohort. Alternatively, this difference may be related to genetic or epigenetic characteristics specific to the Vietnamese population, which could modify susceptibility to mutation. Three of the five recurrent cases harbored the *BRAF*^V600E^ mutation and one carried a *RAS* mutation. The overall mutation frequency in the recurrent group was higher than in the primary group (80% for MAPK pathway mutations and 60% for *BRAF*^V600E^), although the difference did not reach statistical significance. Among the recurrent cases, one involved a maxillary plexiform ameloblastoma initially treated by enucleation, which recurred 5 years after the initial surgery, at which time the recurrent lesion was included for molecular analysis and showed no detectable mutations in the investigated hotspot regions, radiographically, the cortical bone demonstrated expansion without evidence of perforation. Another case was a mandibular mixed-type ameloblastoma (follicular and plexiform patterns) initially treated by enucleation, which recurred 3 years after surgery and harbored a *RAS* mutation. The remaining three recurrent cases were all mandibular ameloblastomas harboring the *BRAF*^V600E^ mutation, including one follicular ameloblastoma treated by segmental resection with late recurrence 15 years after surgery, one luminal unicystic ameloblastoma treated by enucleation with recurrence after 2 years, and one mural unicystic ameloblastoma that recurred 7 years after segmental resection despite microvascular fibular flap reconstruction. Notably, all recurrent mandibular cases were associated with cortical bone perforation at the time of recurrence. Overall, recurrent cases were observed across different histopathological subtypes and occurred following both conservative and radical surgical approaches. The consistent presence of cortical bone changes, showing a progression from bony expansion and cortical thinning to cortical perforation, together with the heterogeneity of molecular mutational profiles and treatment modalities, highlights the biological heterogeneity and locally invasive behavior of ameloblastoma. Given the small number of recurrent cases, meaningful comparison with other studies was limited, and previous reports have also shown no significant difference in recurrence risk between *BRAF*^V600E^-mutant and wild-type cases [39, 40, 46].

Nevertheless, the trend toward higher mutation frequency in recurrent tumors observed in our cohort suggests a potential role of MAPK pathway activation in disease persistence and progression. *BRAF*^V600E^-positive clones may have enhanced proliferative capacity or resistance to conventional treatment, thereby contributing to recurrence. Clinically, these findings highlight the need for vigilant longterm follow-up in patients with MAPK pathway–mutant ameloblastomas, particularly those harboring *BRAF*^V600E^. Moreover, the potential application of *BRAF* inhibitors or combined targeted approaches in recurrent or refractory cases deserves consideration, in light of emerging evidence of therapeutic benefit in other *BRAF*^V600E^-driven tumors.

In this study, MAPK pathway mutations were more frequent in mandibular than maxillary tumors, with the symphysis–body region showing the highest prevalence, whereas *BRAF*^V600E^ mutations were most frequent in the angle-ramus region. Consistent with our findings, previous studies have also reported that *BRAF* mutations predominate in the mandible, while *RAS* mutations are more frequently detected in the maxilla [20].

There is currently limited research on the association between *BRAF* and *RAS* mutations and the radiographic features of ameloblastoma. Therefore, we investigated this relationship across several parameters, including radiolucent pattern, lesion margin characteristics, presence of impacted teeth within the lesion, root resorption, and cortical bone alterations [17, 18, 47]. Our findings revealed that ameloblastomas with a unilocular or soap-bubble radiolucent pattern had a significantly higher frequency of *BRAF*^V600E^ mutations compared with other patterns (Fisher’s exact test, *p* = 0.04). This observation suggests that specific radiographic features may reflect underlying molecular alterations and could serve as useful markers for prognostication. Nevertheless, radiographic appearance is not determined solely by genetic background but may also be influenced by the timing of clinical presentation. Given the slow growing and often asymptomatic nature of ameloblastoma, many patients present only when the tumor has enlarged, which results in more advanced imaging features such as multilocularity, cortical perforation, or extensive root resorption [48–50]. Thus, part of the association between *BRAF*^V600E^ mutations and distinct radiographic patterns in our cohort may have been confounded by symptom duration at diagnosis. Future studies with larger cohorts should adjust for both disease duration and tumor size to validate imaging–genotype correlations and clarify the biological mechanisms by which *BRAF*^V600E^ mutations contribute to bone resorption.

MAPK pathway mutations and *BRAF*^V600E^ mutations were more common in unicystic than conventional ameloblastomas (75% vs. 70%). These findings are consistent with Guimarães [9], González [35], Bonacina [39], and Kelppe [40]. Among histological subtypes, the plexiform subtype showed the highest *BRAF*^V600E^ prevalence (58.8%), consistent with the findings of Shirsat [51]. These parallels suggest possible biological differences among histopathological variants. Notably, the higher frequency of mutations in unicystic ameloblastomas may partly explain their more indolent clinical behavior and responsiveness to targeted therapy compared with conventional forms. Furthermore, the predominance of *BRAF*^V600E^ in the plexiform pattern raises the possibility that distinct molecular mechanisms drive tumor architecture. In the present study, desmoplastic ameloblastoma was identified in 3 of 96 cases. With respect to radiographic features, two cases exhibited a honeycomb radiolucent appearance, whereas one case showed a unilocular radiolucent lesion. Notably, an *NRAS* mutation was detected in one of the two cases with a honeycomb radiolucent pattern. Although the number of desmoplastic ameloblastoma cases was limited and these observations are primarily descriptive, this finding suggests that radiographic phenotypic variability within this rare subtype may reflect underlying molecular heterogeneity. Desmoplastic ameloblastoma is characterized by a predominance of stromal components and lower tumor cellularity compared with other histological subtypes. In the present study, all specimens were histopathologically reviewed prior to DNA extraction to ensure the presence of adequate tumor components for molecular analysis. Histopathological classification in this study followed the WHO Classification of Head and Neck Tumours (2017), which was the reference available at the time the cases were reviewed. The cases were subsequently re-evaluated in the context of the updated WHO Classification of Head and Neck Tumours (5th edition, 2022). Because the diagnostic criteria for ameloblastoma remain essentially unchanged in the updated classification, this is unlikely to affect the interpretation of the present findings [52]. In future studies, systematic assessment of tumor cellularity will be essential to further standardize analytical workflows, better characterize the molecular features of this subtype, and refine the understanding of genotype–phenotype correlations. Taken together, these results support the hypothesis that genetic alterations not only influence tumor initiation but may also contribute to the histopathological phenotype and clinical course of ameloblastomas.

In summary, this study is strengthened by its relatively large sample size, the use of reliable gene sequencing methods, and comprehensive analyses integrating clinical, radiographic, and histopathological features. Nevertheless, several limitations should be acknowledged, including the single-center design conducted in Ho Chi Minh City, the descriptive cross-sectional nature of the study, and the lack of long-term follow-up data on recurrence. Importantly, because molecular testing was performed on FFPE-derived DNA using hotspot-targeted PCR–Sanger sequencing, there remains a potential risk of fixation-related base substitution artifacts and misclassification of rare or previously unreported variants. This approach was not intended for comprehensive exon-wide profiling and may miss uncommon alterations outside the targeted regions, particularly in samples with relatively low tumor cellularity. While PCR-based mutation analysis was considered appropriate for the study objectives, future studies may benefit from incorporating complementary approaches to further optimize mutation detection accuracy, such as DNA damage–mitigation strategies for FFPE samples or the application of next-generation sequencing platforms.

## Conclusions

*BRAF*^V600E^ mutation was the most prevalent genetic alteration in Vietnamese patients with ameloblastoma, occurring in more than half of cases, whereas RAS mutations were uncommon, with one rare variant identified. Furthermore, *BRAF*^V600E^ mutation was significantly associated with sex and radiographic features, being more frequent in females and correlated with distinct radiolucent patterns. Together, these findings support the involvement of MAPK signaling pathway alterations in ameloblastoma and should be regarded as exploratory and hypothesis-generating, providing a basis for future studies to further clarify their biological and potential therapeutic relevance.

## Data Availability

The nucleotide sequence corresponding to the *HRAS* c.22G> A (p.Val8Met, V8M) has been deposited in the European Variation Archive (EVA) under project accession number PRJEB103835 and in GenBank repository under accession number PX605301. All other genetic variants analyzed in this study are previously reported and have corresponding stable public database identifiers (ClinVar, COSMIC, dbSNP). *BRAF* c.1375G> A (p.Val459Met, V459M): COSMIC COSM10284879. *BRAF* c.1333G> A (p.Asp445Asn, D445N): ClinVar RCV00044227; dbSNP rs752429313. *BRAF* c.1397G> A (p.Gly466Glu, G466E): ClinVar RCV000442274; COSMIC COSM453. *BRAF* c.1828T> C (p.Phe610Leu, F610L): dbSNP rs2128998190. *BRAF* c.1798G> A (p.Val600Met, V600M): ClinVar RCV000429286; COSMIC COSM1130. *BRAF* c.1799T> A (p.Val600Glu, V600E): ClinVar RCV000067669; COSMIC COSM476*KRAS* c.35G> T (p.Gly12Val, G12V): COSMIC COSM520; ClinVar RCV000154262. *KRAS* c.50G> A (p.Ser17Asn, S17N): ClinVar RCV002292309; COSMIC COSM51382. *NRAS* c.34G> A (p.Gly12Ser, G12S): ClinVar RCV000421327; COSMIC COSM563. *HRAS* c.31G> A (p.Ala11Thr, A11T): COSMIC COSM4193616. *HRAS* c.205G> A (p.Asp69Asn, D69N): COSMIC COSM6009011.

## Abbreviations

CT: Computed tomography
H &E: Hematoxylin and Eosin
MAPK: Mitogen-Activated Protein Kinase
FFPE: Formalin-fixed paraffin-embedded
SD: Standard deviation
IQR: interquartile range
